# Gait Characteristics of People with Hallux and Forefoot Amputations—A Case Series

**DOI:** 10.3390/jcm14072140

**Published:** 2025-03-21

**Authors:** Frithjof Doerks, Carina Gempfer, Magnus Reulbach, Eike Jakubowitz

**Affiliations:** Laboratory of Biomechanics and Biomaterials, Department of Orthopaedic Surgery, DIAKOVERE Annastift, Hannover Medical School, Anna-von-Borries-Strasse 1-7, 30625 Hannover, Germany

**Keywords:** minor amputation, forefoot amputation, hallux amputation, gait, lower limb amputation

## Abstract

**Background/Objectives:** Minor amputations are increasingly relevant due to a growing proportion of lower limb amputations but remain underrepresented in research. These amputations impair mobility due to altered gait, and biomimetic devices could potentially address this issue. Fundamental research is needed to better understand this pathological gait pattern. The aim of this study is to analyse the holistic gait characteristics of the lower extremity during barefoot walking in individuals with different levels of minor amputations for the first time. **Methods:** Eight young to middle-aged subjects with minor foot amputations (four × hallux; four × forefoot) underwent instrumented gait analysis. Kinematic and kinetic data were acquired barefoot at self-selected gait speeds. Individual gait characteristics were considered relative to the physiological gait represented by the 95% confidence interval of ten unimpaired volunteers. **Results:** Subjects with a minor amputation show reduced walking speed and shorter stride length compared to controls. Sagittal ankle moment and ankle power are lower, with greater deficits in subjects with a forefoot amputation. Proximal joints also show variability, notably reduced knee flexion in subjects with a forefoot amputation and a more flexed hip profile in six subjects. Single-subject frontal plane kinetics also vary. **Conclusions:** Although the subjects with a hallux amputation exhibit smaller deviations in ankle kinetics than the subjects with a forefoot amputation, proximal joint abnormalities are present across cases. These findings highlight the need for a broad range of care to adequately address individual needs.

## 1. Introduction

Although the number of major lower limb amputations has decreased in recent years, the proportion of minor amputations, and their importance, has increased accordingly [1,2,3]. In Germany alone, over 45,000 minor amputations were performed within one year [2]. More than half of all minor amputations involve the forefoot [1,2]. These amputations can be subdivided according to their level: toe amputation, hallux amputation, ray amputation, metatarsophalangeal (MTP) amputation, transmetatarsal (TMT) amputation, Lisfranc amputation, and Chopart amputation [4].

Such amputations limit patients’ mobility due to an impaired gait pattern and can therefore reduce their quality of life [5]. These gait characteristics mainly depend on the amputation level due to different preservations of foot muscles and the remaining foot lever length. Few studies have investigated the baseline condition of people with a minor amputation walking barefoot. Aprile et al. [5] report a lower walking speed and a more variable and reduced lower limb joint range of motion (RoM) for subjects with a first-ray amputation. Forczek-Karkosz et al. [6] find a slower walking speed and a reduced ankle and knee joint RoM but a larger hip joint RoM for a person with bilateral toe amputations. Furthermore, kinetic deficits are also reported for the ankle joint in people with TMT amputation [7]. Compared to subjects with peripheral neuropathy, a population of subjects with partial foot amputation shows an increase in load over the mid-foot and peak plantar pressure during mid-stance [8]. An increased load on the metatarsal head region is observed in subjects with a hallux amputation, which is attributed to a reduced centre of pressure (CoP) velocity in this region [9]. Doi et al. [10] report a lateral CoP shift, limited push-off, impaired balance, and knee pain in a person shortly after a traumatic hallux amputation. To date, there are no motion capture studies conducted either barefoot or with orthopaedic devices on subjects with a hallux amputation. Several orthotic and prosthetic devices aim to maintain stability and compensate for functional deficits [11,12]. Several studies have investigated the walking biomechanics of subjects with a forefoot amputation using orthopaedic devices [7,13,14,15,16,17,18]. The late stance phase, in particular, is impaired with individual devices, and a reduced ability to generate plantar flexor power at the ankle remains [7,13,15]. This is related to limited CoP progression at amputation levels that include metatarsal head loss [14,15,16]. An earlier onset of the hip flexor moment is described as a compensatory pattern [13].

As physiological gait cannot be restored using current devices, further fundamental research on barefoot walking is needed to understand pathological gait and its variability after a minor amputation, so that biomimetic devices can be developed. According to a recent review [4], most studies focusing on gait analysis of subjects with minor amputations use small and heterogeneous populations. The cause, also described in the literature, is the limited local availability of patients coupled with a variety of factors that could influence gait pattern. Furthermore, as reported by Elabd et al. [8], systematic diseases underlying the cause of amputation and other factors, such as the exact level of amputation, age, and the time since amputation, can influence the biomechanical parameters.

Due to the variety of factors that influence gait pattern, there is a risk of drawing incorrect conclusions when grouping and investigating small cohorts. Initially, a prospective study was planned, but the first data evaluation revealed such great variability, and the acquisition of a homogeneous cohort could not be achieved, that the data were analysed in the form of a case series.

The aim of this study is to analyse the holistic kinematic and kinetic parameters of the lower extremity in subjects with different levels of amputation. Taking into account the importance of the hallux for energy storing and releasing mechanisms [19], its role in speed generation [20], and the lack of corresponding data in the literature, this study integrates the analysis of kinematic and kinetic data of individuals with a hallux amputation for the first time. By comparing the compensation patterns of people with a hallux amputation both with the compensation patterns of other minor amputation levels and with physiological walking, it can be determined whether a grouping with more proximal minor amputation levels is appropriate. Furthermore, implications for the clinical care of specific patient groups can be derived.

## 2. Materials and Methods

### 2.1. Participants

For the initially planned prospective study, patients with a minor amputation were recruited through clinics, orthopaedic shoe technicians, podiatrists, conferences, and social media. Participants were informed about the study procedures by the principal investigator (F.D.) and were screened for inclusion and exclusion criteria prior to participation. Given the challenges in patient recruitment and the high inter-subject variability, this study investigates eight subjects (Table 1) with a unilateral foot minor amputation in the form of a case series. The inclusion criteria were written consent to participate, a minimum age of 18 years, and a unilateral minor foot amputation. The exclusion criteria included additional musculoskeletal diseases affecting gait, a more proximal amputation level than the Lisfranc level, and a limited ability to walk without aids. A healthy group without amputation consisting of ten volunteers selected from the gait laboratory database (5 F; age: 26.2 ± 2.4 years; weight: 72.6 ± 15.4 kg; height: 175.4 ± 13.1 cm) served as a reference data set representing the unaffected gait.

This study was approved by the local ethics committee of the Hannover Medical School (#10522) in accordance with the Declaration of Helsinki, and all subjects gave written informed consent prior to participation.

### 2.2. Instrumentation and Experimental Protocol

First, anthropometric data and amputation-related information were collected for each subject. Infrared retroreflective markers (12 mm) were attached to the skin of each subject according to the Plug-in-Gait model [21]. For subjects with a forefoot amputation, the toe marker was placed on the dorsum of residuum and the height of the heel marker was aligned accordingly. The subjects were asked to walk a 10 m walkway in the gait laboratory several times without shoes at a self-selected walking speed, until seven double force plate strikes were recorded. Kinematics were acquired with a 200 Hz motion capture system consisting of twelve MX infrared cameras (Vicon Motion System Ltd., Oxford, UK) for three subjects and the healthy controls and with an equivalent system consisting of twelve Miqus infrared cameras (Qualisys AB, Gothenburg, Sweden) for five subjects. Ground reaction forces (GRF) were measured with 1000 Hz by two force plates (Type BP400600, AMTI, Watertown, MA, USA) embedded in the walkway.

### 2.3. Data Analysis

All the data were processed with the software Vicon Nexus 1.8.5. Time-distance parameters were determined and averaged for seven trials. Descriptive statistics included means ± SD. The kinematic and kinetic output parameters were time normalised and averaged using an algorithm written in MATLAB (The MathWorks, R2021b, Natick, MA, USA). The kinematic and kinetic data of the control group were averaged for the left and right extremities. However, the ipsilateral side is reported for the subjects with a minor amputation. Kinetic is reported as external joint moments. For the kinematic data, a RoM analysis was additionally performed to compensate for possible offset errors due to critical marker placements, as it focuses on relative rather than absolute values. The gait characteristics of each subject were considered relative to the physiological gait represented by the control group’s 95% confidence interval (CI). Since this study is a case series focusing on the gait of subjects with different levels of minor amputation, the control group serves only as a visual reference for physiological walking, and no statistical analysis is performed. The Gait Variable Scores (GVSs) were calculated for the presented kinematic parameters according to Baker et al. [22] and calculated in a modified Gait Profile Score (mGPS).

## 3. Results

### 3.1. Time–Distance Parameter

Subjects with a minor amputation generally have a lower self-selected walking speed than the healthy control group (Table 2). Stride length is also shortened compared to the controls. TMT2 is an exception and has a walking speed comparable to a healthy person’s but with a reduced stride length. Apart from TMT2, the subjects with a hallux amputation have a higher walking speed than the subjects with a forefoot amputation. Whereas there are only small differences in stride length between the ipsilateral and contralateral side, the relative proportion of the stance phase varies in some cases. Two subjects (HAL2 and HAL4) have symmetrical relative times of foot off between the sides. Other subjects have a shorter relative stance phase on the ipsilateral side, with the greatest difference in the person with a Lisfranc amputation (LIS1).

### 3.2. Kinematic Data

**Figure 1:**
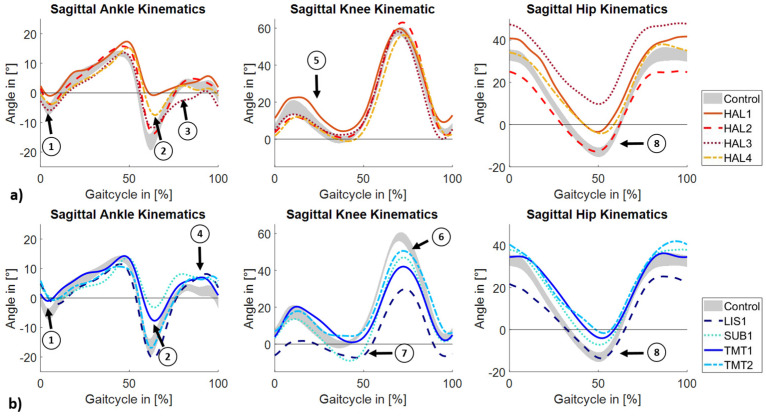
Mean sagittal joint angles of the ipsilateral side for (**a**) subjects with a hallux amputation and (**b**) subjects with a forefoot amputation and the 95% CI of the control group.

While the initial plantar flexion peak is hardly prominent in all subjects with a forefoot amputation and one person with a hallux amputation (HAL1), it is present in the other subjects with a hallux amputation (tag 1, Figure 1a,b). In four subjects (HAL1, HAL4, SUB1, TMT1), a reduced plantar flexion angle of less than 10° at the end of the stance phase is evident (tag 2). HAL3 has a more plantar-flexed profile during the swing phase compared to the control group (tag 3). Subjects with a forefoot amputation, on the other hand, have greater dorsiflexion in the terminal swing phase (tag 4). The sagittal movement pattern of the knee joint of the subjects with a hallux amputation is comparable to the control group. The maximum deviation of the RoM from the control group is 4° in HAL1, who also shows greater knee flexion during the stance phase (tag 5). A significant reduction in knee flexion peak during the swing phase can be observed in subjects with a forefoot amputation (tag 6). In contrast, an ambiguous pattern can be seen in terminal stance with two subjects (LIS1, SUB1; tag 7) with increased knee extension compared to the healthy group. RoM is most reduced in LIS1 at 38° compared to 60° of the controls. A large variability is observed in hip joint movement. While six subjects show a more flexed hip profile during the stance phase, the extension peak in two subjects (HAL2, LIS1; tag 8) is comparable to that of the healthy subjects. One hallux amputee (HAL3) has significant deviations from the physiological hip movement during the entire gait cycle with an absence of complete hip extension. RoM is most reduced in subjects HAL2, HAL3, and LIS1, with a range of less than 40°. While six subjects have similar mGPS independent of the amputation level, in two subjects the mGPS is above 10° (Table 3).

### 3.3. Kinetic Data

**Figure 2:**
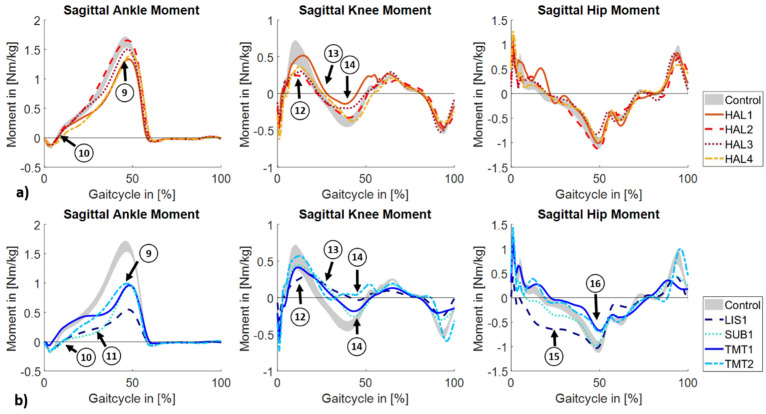
Mean external sagittal joint moments for (**a**) subjects with a hallux amputation and (**b**) subjects with a forefoot amputation and the 95% CI of the control group.

Peak dorsal flexion moment is reduced in six subjects compared to the control group (tag 9, Figure 2a,b). While the peak in the subjects with a hallux amputation is slightly reduced, the peak in subjects with a TMT amputation is only about 60% of the value in healthy individuals and, in the person with a Lisfranc amputation, only 34%. In four individuals (HAL4, LIS1, SUB1, TMT1; tag 10), a prolonged plantar flexion moment is observed in loading response, and, in two of them, a slower increase in the dorsal flexion moment during mid-stance can be seen (LIS1, SUB1; tag 11). Initial knee flexion moment peak is reduced in three subjects (HAL2, HAL3, LIS1; tag 12). Furthermore, the period of knee flexion moment is prolonged in all subjects with a forefoot amputation and one person with a hallux amputation (HAL1; tag 13). In the terminal stance phase, the knee extension moment is limited in six subjects, with a nearly complete absence in two of them (LIS1, TMT2; tag 14). While subjects with a hallux amputation show a physiological hip moment pattern, the hip moment pattern of the subjects with a forefoot amputation is ambiguous. A premature and excessive hip extension moment is observed in two subjects (LIS1, SUB1; tag 15). In contrast, two subjects (TMT1, TMT2; tag 16) show a limited hip extension moment peak.

**Figure 3:**
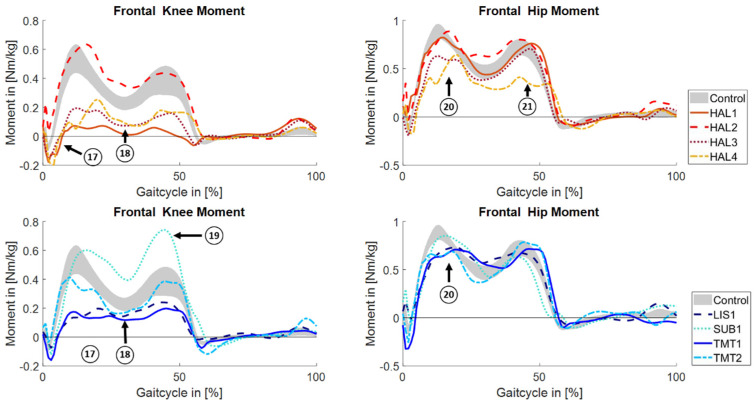
Mean external frontal joint moments for subjects with a hallux amputation and subjects with a forefoot amputation and the 95% CI of the control group.

Deviations from the control group also exist in the frontal plane kinetics (Figure 3). A prolonged knee abduction phase is observed in six subjects (tag 17). While two subjects (HAL2, TMT2) show only slight deviations from the control group, knee adduction moment is reduced in five subjects (tag 18) during almost the entire stance phase and increased in one person (SUB1, tag 19). The first peak of the hip adduction moment is reduced in two subjects with a hallux amputation and three subjects with a forefoot amputation (tag 20). The second peak is only markedly reduced in HAL4 (tag 21).

**Figure 4:**
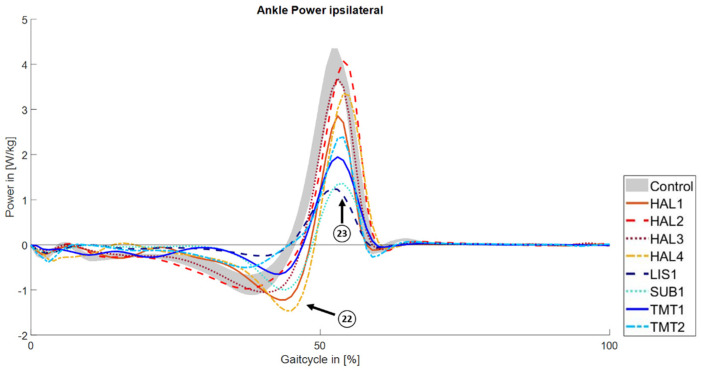
Mean ankle power for subjects with a minor amputation and the 95% CI of the control group.

In some cases, a prolonged phase of power absorption at the ankle is observed (Figure 4, HAL1, HAL4, SUB1; tag 22). The power generation peak is reduced in six subjects (tag 23). While two subjects with a hallux amputation have comparable values to the healthy control group, the peak in HAL1 and HAL4 is reduced by 27% and 6%, respectively. Subjects with a forefoot amputation show greater impairments with the maximum reduction of 68% observed in the person with a Lisfranc amputation (LIS1).

## 4. Discussion

This series is the first to analyse barefoot walking in subjects with different levels of minor amputation. As hypothesised and summarised in a recent review [4], biomechanical data have a high inter-subject variability and emphasise the need for a case-by-case analysis.

### 4.1. Time-Distance Parameter

With the exception of one, subjects with a minor amputation have a lower walking speed than the controls in this study. On the other hand, a shorter stride length is observed in all subjects. These findings are consistent with previously published data [5,6]. Given the positive correlation between stride length and quality of life scores in individuals with a minor amputation reported by Aprile et al. [5], the quality of life of these patients may be limited by their worsened biomechanical data. In this study, subjects with a hallux amputation walk at a faster speed than three of the subjects with a forefoot amputation. Furthermore, two subjects with a hallux amputation have symmetrical relative stance phase proportions between the ipsilateral and contralateral side. Similar results are found by Aprile et al. [5] for subjects with a first-ray amputation. All other individuals have greater stance phase proportions on the contralateral side, presumably due to reduced residual length on the affected side and for improved stability.

### 4.2. Methodological Challenges

Due to the difficulty of patient acquisition and the frequent concomitant symptoms of obesity, no exclusion criteria were made for weight. Knee joint kinematics for LIS1 and hip joint kinematics for HAL3 show clearly unexpected kinematic profiles also resulting in higher GVS and mGPS. Even if Pau et al. [23] associate similar tendencies with the biomechanical influence of obesity, this is more likely explained by the difficulty in precisely placing the ASIS (anterior superior iliac spines) markers, resulting in inaccurate determination of the hip joint centre. This is emphasised by observation of the upright standing position of HAL3, in which hip flexion due to tilting of the pelvic segment can also be observed. Nevertheless, motion capture with standard marker sets is still the most commonly used method for capturing the kinematics of such populations [24], and we also analysed RoM values to take possible offset errors into account.

### 4.3. Sagittal Plane Biomechanics

Kinematic characteristics of the ankle and knee joint of subjects with a hallux amputation during mid-stance generally correspond to the physiological pattern. An excessive ankle dorsiflexion with a flexed knee during the stance phase is observed in one subject (HAL1), indicating weakness of the m. soleus to control tibia progression [25]. Greater variability in the kinematics during mid-stance is seen in subjects with a forefoot amputation. In accordance with the literature [5,6,7,13,15], the pre-swing phase, including the push-off, is the most affected due to the loss of lever length and muscular attachments.

Plantar flexion peak is limited in some individuals. Inter-subject variability is high, and there is no correlation with amputation level, cause of amputation, or age. As previously reported [7,13,17], sagittal ankle joint moments are also severely restricted. There are substantial differences between subjects with a hallux amputation and subjects with a forefoot amputation according to the remaining foot length. Not only is the peak in the subjects with a forefoot amputation limited, but the progression of the dorsal extension moment is also delayed in four individuals (HAL1, LIS1, SUB1, TMT2). The greatest deviations can be seen in the subject with the shortest residual (LIS1), which emphasises the influence of the remaining foot lever and the corresponding muscle preservation [26]. Peak ankle power generation is also limited in most subjects. This biomechanical parameter is usually reported according to a recent review [4], and the abnormality of individuals with a minor amputation is attributed to muscular weakness of the plantar flexors and atrophy in addition to the lack of a forefoot lever.

An interesting finding can be identified by combining the kinematic and kinetic parameter of the ankle. The sagittal kinematic and kinetic patterns of the subjects with a hallux amputation HAL2 and HAL3 correspond to those of the healthy group and can generate physiological ankle power. As these are the two individuals with the least time since amputation, it can be hypothesised that compensatory strategies and an unused remaining forefoot lever leads to atrophy over time as reported for major amputations [27]. However, a posterior shift of the loads to the mid-foot, as described by Elabd et al. [8], can also be responsible for the limited moments. The prolonged knee flexion moment and the diminished knee extension moment observed during the stance phase in certain individuals in the present study support this assumption. The GRF vector remains situated posterior to the knee joint centre for an extended duration compared to healthy subjects, a phenomenon that can be correlated to the restricted centre of pressure progression observed in subjects with TMT and Lisfranc amputations reported by Dillon and Barker [15]. Elabd et al. [8] posit that a posterior shift of the load is a mechanism to compensate for insufficient forefoot support, potentially leading to a greater foot progression angle or medial longitudinal arch collapse. At the same time, a longer external knee flexion moment may necessitate increased engagement of the quadriceps muscles, resulting in elevated energy expenditure. As Aprile et al. [5] conclude, the inability to generate plantar flexor power at the ankle affects the kinematic behaviour of all lower limb joints. The current study observed a more flexed hip profile and reduced RoM in six out of eight subjects. While Aprile et al. [5] cannot clarify whether the observed behaviour was due to underlying diabetes or the amputation, the pattern is observed for different causes of amputation in the current study. Significant differences between subjects with a hallux amputation and subjects with a forefoot amputation can be observed in the swing phase. While the subjects with a hallux amputation have physiological knee flexion, it is reduced in all subjects with a forefoot amputation. Similar results are reported by Aprile et al. [5] for subjects with a first-ray amputation walking barefoot. Given that people with a minor amputation do not show reduced knee flexion when wearing devices [15,28], this may be an automatic energy-saving mechanism, as the shorter foot length requires less flexion to assure foot clearance of the floor.

### 4.4. Frontal Plane Biomechanics

Abnormalities can be observed not only in the sagittal but also the frontal plane. Considering the correlation between mediolateral CoP shift and knee adduction moment [29], the results of this series are particularly interesting. Three subjects with a hallux amputation, i.e., with an asymmetric amputation with preservation of the lateral side, show similar patterns with lower adduction moments. On the other hand, the person with an asymmetrically longer medial residual limb (SUB1) shows increased adduction moments. This is relevant in view of the conclusion of D’Souza et al. [30] regarding an association between frontal plane biomechanics and the odds for the progression of tibiofemoral and hip osteoarthritis.

### 4.5. Implications for Novel Device Concepts

The current study demonstrates the high variability in the effects of a minor amputation on biomechanical parameters. As described in the theory [26], the different amputation levels lead to different gait deviations. Even if subjects with a hallux amputation can generate physiological ankle power in contrast to more proximal amputations, compensatory gait abnormalities are possible. Despite TMT2 having a walking speed comparable to healthy people, compensatory mechanisms can be seen in the knee and hip, which can lead to further musculoskeletal problems in the long term. The modified Gait Profile Score is in a similar range to people with major amputations [31], and so more attention should be paid to minor amputations in studies. Since existing orthopaedic devices cannot fully compensate for the deficits in gait [7,14,15,16], innovative devices should be developed. Insights drawn from the present findings suggest several implications for such devices. Already in mid-stance, the device should actively facilitate forward progression of the GRF vector to prevent abnormal moments in the proximal joints from occurring. At the same time, the terminal portion of the residual limb should be effectively protected to facilitate forefoot loading, thereby allowing the residual forefoot lever to be optimally utilised by the musculature. The incorporation of energy storage and release mechanisms becomes crucial to support the push-off phase and compensate for deficiencies in power generation.

### 4.6. Limitations and Future Perspectives

Due to the correlation between gait speed and kinematic and kinetic parameters [32], it is possible that the results were also influenced by the different self-selected speeds. In future, standardised speeds should additionally be investigated. In contrast, no relevant age-related differences in self-selected speed, joint motion, or moments were expected between the young control group and the middle-aged patient group [33,34].

In future studies, multi-centre studies should be initiated to overcome the challenges described for acquiring homogeneous cohorts with sufficient group size to integrate a statistical analysis. The lack of surgical reports and clinical examinations regarding the exact description of remaining structures limits the conclusions. It cannot be completely ruled out that the different causes of amputation, such as vascular diseases, may have influenced the results, so that subgroups with the same level of amputation but different causes should also be examined in future studies. However, this study showed similar trends in cases with the same amputation level but different causes. Future studies should also measure lower extremity muscle activity, pressure distribution, and upper body movement to further understand the complex mechanisms. Given that the conventional calculation of ankle power does not consider the contribution of the foot to the push-off [35], it is likely that the power generated by the entire foot–ankle complex will also deviate more from the controls in subjects with a hallux amputation. In addition, a follow-up of subjects HAL2 and HAL3 would be useful to test the hypotheses of the temporally altered plantar flexor power generation ability.

The present study provides valuable insights into the gait abnormalities observed in subjects after minor amputation. As this study shows, young people are also affected by such amputations and the resulting effects on gait. Further research should be carried out to facilitate their social participation and mobility.

## 5. Conclusions

This study emphasises the individual effects of a minor amputation on walking. Furthermore, it shows the high inter-subject variability and heterogeneity of the group of subjects with minor amputations stressing that a grouping of patients should only be made if there is a high degree of agreement on amputation-specific characteristics. As substantial gait abnormalities are observed in some cases, research should be intensified to create a broader range of orthopaedic devices to meet individual needs. Energy storing and releasing devices should support the push-off phase, thereby encouraging proactive utilisation of the remaining foot length and mitigating the risk of muscle atrophy. Even if subjects with a hallux amputation show minor deviations from physiological walking, they should also be fitted with adequate devices to avoid compensatory mechanisms and optimise their overall mobility and well-being.

## Figures and Tables

**Table 1:** Subject-specific data.

Subject	Level of Amputation	Sex	Age (y)	Weight (kg)	Height (m)	Residual Length (% Contralateral Foot Length)	Cause of Amputation	Time since Amputation (y)
HAL1	Hallux	F	52	66.8	1.57	95.1	Diabetes Type II	6
HAL2	Hallux	M	59	70.4	1.80	87.7	Diabetes Type I	<1
HAL3	Hallux	F	41	83.7	1.72	95.0	Diabetes Type I	<1
HAL4	Hallux	F	31	58.9	1.71	91.7	Deformity	-
LIS1	Lisfranc	F	47	110.7	1.74	58.8	Embolisms	6
SUB1	Subtotal TMT *	F	22	75.8	1.59	75.0	Trauma	19
TMT1	TMT	F	56	54.1	1.59	62.2	Trauma	35
TMT2	TMT	M	29	73.3	1.70	73.7	Trauma	4

* Asymmetrical resection of the metatarsals with greater metatarsal I preservation.

**Table 2:** Time–distance parameter ± SD for the individual subjects.

Subject	Walking Speed [m/s]	Stride Length Ipsilateral [m]	Stride Length Contralateral [m]	Foot Off Ipsilateral [%]	Foot Off Contralateral [%]
Controls	1.41 ± 0.1	1.44 ± 0.1		58.93 ± 1.18	
HAL1	1.23 ± 0.05	1.15 ± 0.03	1.12 ± 0.01	58.32 ± 0.66	60.50 ± 0.27
HAL2	1.23 ± 0.03	1.30 ± 0.03	1.32 ± 0.03	60.75 ± 0.55	60.85 ± 0.69
HAL3	1.19 ± 0.03	1.20 ± 0.01	1.17 ± 0.01	59.58 ± 0.79	61.00 ± 0.80
HAL4	1.26 ± 0.01	1.29 ± 0.01	1.27 ± 0.01	59.57 ± 0.92	59.51 ± 0.81
LIS1	0.84 ± 0.04	1.09 ± 0.04	1.07 ± 0.03	58.18 ± 1.33	66.10 ± 1.00
SUB1	1.15 ± 0.03	1.17 ± 0.03	1.17 ± 0.02	59.38 ± 0.60	64.11 ± 0.93
TMT1	1.03 ± 0.03	1.07 ± 0.01	1.06 ± 0.01	59.09 ± 0.66	61.52 ± 0.57
TMT2	1.39 ± 0.03	1.29 ± 0.02	1.30 ± 0.02	57.75 ± 0.59	61.03 ± 0.63

**Table 3:** Gait Variable Scores (GVSs) and modified Gait Profile Score (mGPS) for the individual subjects.

Subject	GVS_Ankle_	GVS_Knee_	GVS_Hip_	mGPS
HAL1	5.8°	5.0°	8.7°	6.7°
HAL2	2.3°	3.2°	5.6°	4.0°
HAL3	3.0°	3.2°	18.6°	11.0°
HAL4	4.1°	3.9°	5.8°	4.7°
LIS1	3.6°	16.9°	7.0°	10.8°
SUB1	6.0°	8.0°	4.9°	6.4°
TMT1	3.9°	8.1°	7.1°	6.6°
TMT2	2.9°	4.6°	8.5°	5.8°

## Data Availability

The datasets used and analysed in the current study are available from the corresponding author on reasonable request.

## Abbreviations

RoM	range of motion
MTP	metatarsophalangeal
TMT	transmetatarsal
GRF	ground reaction force
CI	confidence interval
GVS	Gait Variable Score
mGPS	modified Gait Profile Score
